# microRNAs participate in gene expression regulation and phytohormone cross-talk in barley embryo during seed development and germination

**DOI:** 10.1186/s12870-017-1095-2

**Published:** 2017-09-06

**Authors:** Bin Bai, Bo Shi, Ning Hou, Yanli Cao, Yijun Meng, Hongwu Bian, Muyuan Zhu, Ning Han

**Affiliations:** 1 Key Laboratory for Cell and Gene Engineering of Zhejiang Province, Institute of Genetics and Regenerative Biology, College of Life Sciences, Zhejiang University, Zhejiang, Hangzhou 310058 China; 2 College of Life and Environmental Sciences, Hangzhou Normal University, Zhejiang, Hangzhou 310036 China

**Keywords:** Barley (*Hordeum vulgare*), Microrna, Seed development, Germination, Embryo, Auxin response, Abscisic acid, Gibberellic acid

## Abstract

**Background:**

Small RNA and degradome sequencing have identified a large number of miRNA-target pairs in plant seeds. However, detailed spatial and temporal studies of miRNA-mediated regulation, which can reflect links between seed development and germination are still lacking.

**Results:**

In this study, we extended our investigation on miRNAs-involved gene regulation by a combined analysis of seed maturation and germination in barley. Through bioinformatics analysis of small RNA sequencing data, a total of 1324 known miRNA families and 448 novel miRNA candidates were identified. Of those, 16 known miRNAs with 40 target genes, and three novel miRNAs with four target genes were confirmed based on degradome sequencing data. Conserved miRNA families such as miR156, miR168, miR166, miR167, and miR894 were highly expressed in embryos of developing and germinating seeds. A barley-specific miRNA, miR5071, which was predicted to target an *OsMLA10-like* gene, accumulated at a high level, suggesting its involvement in defence response during these two developmental stages. Based on target prediction and Kyoto Encyclopedia of Genes and Genomes analysis of putative targets, nine highly expressed miRNAs were found to be related to phytohormone signalling and hormone cross-talk. Northern blot and qRT-PCR analysis showed that these miRNAs displayed differential expression patterns during seed development and germination, indicating their different roles in hormone signalling pathways. In addition, we showed that miR393 affected seed development through targeting two genes encoding the auxin receptors TIR1/AFBs in barley, as over-expression of miR393 led to an increased length–width ratio of seeds, whereas target mimic (MIM393)-mediated inhibition of its activity decreased the 1000-grain weight of seeds. Furthermore, the expression of auxin-responsive genes, abscisic acid- and gibberellic acid-related genes was altered in miR393 misexpression lines during germination and early seedling growth.

**Conclusions:**

Our work indicates that miRNA-target pairs participate in gene expression regulation and hormone interaction in barley embryo and provides evidence that miR393-mediated auxin response regulation affects grain development and influences gibberellic acid and abscisic acid homeostasis during germination.

**Electronic supplementary material:**

The online version of this article (10.1186/s12870-017-1095-2) contains supplementary material, which is available to authorized users.

## Background

Seed development and germination are two critical developmental phase transitions during a plant life cycle. As for most flowering plants, seed maturation and germination are separated by a period of quiescence, which is termed as dormancy [1]. Only after breaking dormancy, the quiescent embryo is able to germinate after imbibition. On the other hand, there is a close connection between these two processes. Genome-wide expression analysis and comprehensive metabolite studies revealed that initial protein synthesis during germination uses pre-existing mRNAs stored in mature dry seed, and metabolic preparation for germination is already initiated during the late stages of seed maturation [2, 3].

Most seeds are composed of three basic parts: embryo, endosperm and seed coat. The embryo encloses fundamental elements and patterns necessary for the new plant to develop after germination, whereas the endosperm provides nutrition for the embryo to use during germination [4]. Studies at the physiological and molecular levels have demonstrated that seed maturation and germination involve tight gene regulation and hormone control so that continuous interchange of signals between the different compartments of seeds is ensured [4–7]. Two hormones, abscisic acid (ABA) and gibberellic acid (GA), play important roles in determining the physiological state of the seed and regulating the development and germination process. The level of ABA peaks during seed maturation and dormancy, whereas GA level increases during imbibition and remains high during germination and postembryonic growth [1]. However, it seems to not be the absolute concentration but the balance of ABA and GA that determines the two events. In addition, another hormone, auxin, has also been proven to be fundamental in the first step of seed development, as well as for the determination of embryo structure and size [4, 8]. In *Arabidopsis*, it is reported that auxin plays a role in seed dormancy and germination through its crosstalk with other hormones such as ABA, indicating a coordinating network of auxin and ABA signalling in this important process [9].

An important mechanism controlling gene expression during seed development and germination is exerted by microRNAs (miRNAs) [10, 11]. miRNAs are a class of endogenous non-coding small RNAs (sRNAs) approximately 21–22 nt in length, which post-transcriptionally regulate gene expression by targeting mRNA for cleavage or translation suppression [12]. High throughput small RNA sequencing coupled with degradome analysis allows identification of conserved and novel miRNA-target pairs in seed of rice, maize, barley and *Brassica napus* [13–20]. Increasing evidence shows that miRNAs play crucial roles in diverse aspects of biological and metabolic processes in seeds including embryogenesis, pattern establishment, seed dormancy, seed germination and early seedling growth. Mutants lacking components of miRNA biogenesis and/or processing displayed severely abnormal seed development or even lethality [21, 22]. It was reported that miR172 affected seed size and yield through targeting several *APETALA2*-like transcription factors [23]; the *mir159ab* double mutant exhibited reduced seed size and altered seed shape [24]. miR159 and miR160 affected the process of germination by regulating ABA sensitivity. Transgenic plants which overexpressed a miR160-resistant form of *ARF10* were hypersensitive to ABA, indicating a point of cross-talk between ABA and auxin in imbibed seeds [25].

Barley (*Hordeum vulgare* L.) is one of the most important cereal crops worldwide, ranking fourth in terms of production and is widely used for brewing and animal feed. Seed maturation and germination are closely associated with crop yield and processing quality of barley grains. Many barley miRNAs have been identified through small RNA sequencing using samples of leaf, seedling, root, and seeds at early development stages [20, 26–30]. However, for the embryo, which is an important tissue affecting seed development and germination, a complete miRNA expression profile analysis has not yet been performed. The purpose of this study was to discover embryo-specific expression of miRNAs and their potential targets and provide vital clues for further and detailed functional studies of barley miRNAs.

## Methods

### Plant materials

Barley (*Hordeum vulgare* L. ‘Golden Promise’), obtained from the Australian Centre for Plant Functional Genomics at the University of Adelaide, was used in this study. The plants were grown from October to May in soil under natural conditions at the Agricultural Experiment Station of Zhejiang University, Hangzhou, Zhejiang Province, China. Immature embryos at 10 days post anthesis (10 DPA) were dissected from spikes. To collect embryos at 1 DAG (days after germination) and 5 DAG, seeds were surface sterilized, rinsed, and then placed on moist filter papers in 9-cm culture dishes and kept at 4°C in the dark for 48 h. They were then grown for 1 d and 5 d in containers with 0.1 mM CaCl_2_ solution (pH 5.8) at 24°C under a 16 h light/8 h dark photoperiod in a controlled climate chamber.

### Small RNA sequencing and identification of conserved and novel miRNAs in barley

**Fig. 1:**
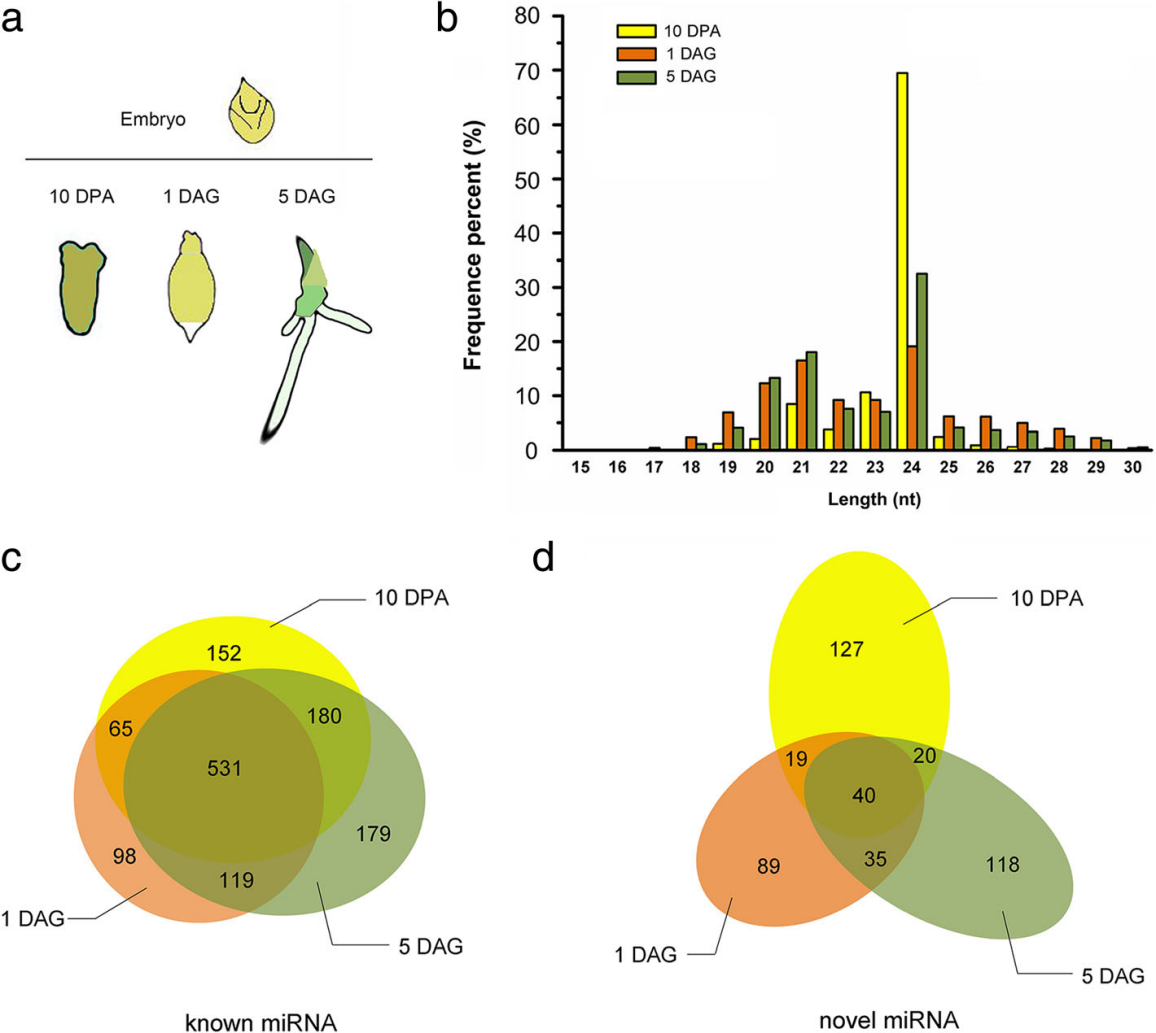
Overview of small RNAs expressed in barley embryos during seed maturation and germination. **a**: Stages of grain development and germination used for sRNA sequencing; **b**: Length distribution of sRNAs in three sequencing samples; **c**-**d**: Venn diagram showing common and specific miRNAs among three samples. c: known miRNAs; d: novel miRNAs

Total RNA from at least 3 g embryos for each sample, as described in Fig. 1a, was isolated using TRIzol Reagent (Invitrogen, USA) according to the manufacturer’s instructions. RNA extracted from three biological replicates was pooled to constitute each sample for small RNA sequencing. The RNA quality was examined using gel-electrophoresis (28S:18S ratio > 1.5) and a Bioanalyzer (Agilent2100, RIN ≥ 8.0). The sRNA libraries were then constructed following the standard protocol used at The Beijing Genomics Institute (BGI, Shenzhen, China). The qualified total RNA samples were gel purified and fragments within the length range (18 nt to 30 nt) were selected to build a library. Then, two kinds of adapters were ligated to each end of the resulting fragments. The prepared RNA was amplified by reverse transcription PCR (RT-PCR) and RT-PCR products were then loaded on a Hiseq2000 platform for sequencing. The raw data from Hiseq sequencing went through data cleaning analysis, which included the elimination of low-quality tags and 5′ adaptor contaminants, to obtain sufficiently clean tags. After filtering, sRNAs with sizes ranging from 18 to 30 nt were aligned to the barley genome (http://plants.ensembl.org/Hordeum_vulgare/Info/Index), Genbank, and Rfam (http://rfam.xfam.org/), and the sequences matching known barley rRNAs, tRNAs, snRNAs, and snoRNAs were discarded. Unannotated small RNA tags were then aligned with miRBase 21 (http://www.mirbase.org/), and those which could be aligned to an miRNA precursor in miRBase with no mismatch, or have at least a 16-nt overlap aligning to a known barley miRNA in miRBase, were termed as known miRNAs. If there was no miRNA information for the species in miRBase, the candidates which could be aligned to any miRNA precursor or mature miRNA from all plants in miRBase, allowing two mismatches or free gaps and with a predicted precursor, were regarded as known miRNAs. After alignment, small RNA tags corresponding to exons and introns of mRNA (to find degraded fragments of mRNA) and the unannotated tags were used for novel miRNA prediction through the Mireap software (http://sourceforge.net/projects/mireap/) developed by BGI. The secondary structures of all identified and potential pre-miRNAs in the barley genome were predicted by using RNAfold software (http://rna.tbi.univie.ac.at/cgi-bin/RNAWebSuite/RNAfold.cgi). The minimal folding energy index (MFEI) of the novel miRNAs was set to be equal or greater than 0.9.

Differentially expressed miRNAs were identified by plotting log2-ratio figures and scatter plots. The expression of miRNAs was normalized according to the expression of transcript per million (TPM). For each sample, TPM = (actual miRNA count/total count of clean reads) × 10^6^. The fold-change in miRNA expression was calculated as fold change = log2 (treatment/control). The differentially expressed sequence counts were analysed by the online service IDEG6 (http://telethon.bio.unipd.it/bioinfo/IDEG6_form/). We considered a fold change of at least 2 and a *P* value cut-off of *P* < 0.00001 as an indication of significant change and considered the given miRNA as a specific development stage-associated miRNA. The chosen potential pre-miRNA sequences obtained from the results of Blastall were subjected to the Zuker folding algorithm for in silico secondary structure generation via the web-based computational software MFOLD.

### Target prediction and degradome sequencing data analysis

The prediction of putative targets for identified miRNAs was performed with Mireap and psRNATarget (http://plantgrn.noble.org/psRNATarget). The following modified parameters for the Mireap software analysis were used: (1) No more than four mismatches between sRNA and target (G-U bases count as 0.5 mismatches); (2) No more than two adjacent mismatches in the miRNA/target duplex; (3) No adjacent mismatches in positions 2–12 of the miRNA/target duplex (the 5′ end of the miRNA); (4) No mismatches in positions 10–11 of miRNA/target duplex; (5) No more than 2.5 mismatches in positions 1–12 of the of the miRNA/target duplex (the 5′ end of miRNA); and (6) The minimum free energy (MFE) of the miRNA/target duplex should be greater than or equal to 75% of the MFE of the miRNA bound to it’s perfect complement. Degradome analyses were based on the barley degradome sequencing dataset (Accession number SRR513549, SRR513550 and SRR513551) downloaded from NCBI. The miRU algorithm with ‘default parameters’ was used in this study [31].

Gene ontology (GO) is an international standardized classification system for gene function, which supplies a set of controlled vocabulary to comprehensively describe the property of genes and gene products. There are three ontologies in GO: molecular function, cellular component and biological process. This method first maps all target gene candidates to GO terms in the database (http://www.geneontology.org/), calculating gene numbers for each term. It then uses a hypergeometric test to find significantly enriched GO terms in target gene candidates, compared to the reference gene background. Then, the GO functional analysis of the putative targets was performed by WEGO (http://wego.genomics.org.cn/cgi-bin/wego/index.pl). GO terms with a *P*-value <0.05 were considered to be significantly enriched.

To facilitate the understanding of the biological functions of the target gene candidates, Kyoto Encyclopedia of Genes and Genomes (KEGG) pathway analysis was used to identify significantly enriched metabolic pathways or signal transduction pathways in target genes compared with the whole reference gene background. The calculating formula was the same as that in the GO analysis.

### Northern blot analysis of miRNA expression

Total RNA was extracted using TRIzol reagent (Invitrogen, USA). For RNA gel blots, 30 μg of total RNA was separated on a 17% polyacrylamide gel containing 7 M urea, blotted to HyBond-N+ membranes (Roche, Germany) and fixed by UV crosslinking. Blots were hybridized using digoxigenin end-labelled locked nucleic acid (LNA) oligonucleotide probes designed against miRNAs. The miRNA level was standardized with that of rRNA. Sequences of the oligonucleotide probes are listed in Additional file 1: Table S5.

### Real-time quantitative RT-PCR analysis of miRNAs and their target levels

Total RNA from barley embryos and seedling was extracted using TRIzol reagent (Invitrogen) and 1 μg was used for first-strand cDNA synthesis using Oligo(dT) primers and a Super-Script III RT kit (TaKaRa, Dalian, China). Quantitative real-time PCR (qRT-PCR) was performed on the Mastercycler ep realplex2 system (Eppendorf, Hamburg, Germany) using a SYBR PrimeScript RT reagent Kit (Perfect Real Time, TaKaRa). Relative transcript levels were calculated with the ΔΔCt method and the *ACTIN* or *UBQ* gene was used as a reference. Quantification of mature miRNA level was carried out through a poly(A)-based real-time PCR approach using Mir-X™ miRNA First Strand Synthesis Kit (Clontech Laboratories, Inc., Cat. #638315) and the miRNA level was standardized with U6. Sequences of primers are listed in Additional file 1: Table S5. The data shown were obtained from three biological replicates.

### Validation of target genes through RLM-5′ RACE

RNA ligase-mediated 5′ rapid amplification of cDNA ends (RLM 5′-RACE) was performed using a SMART TM RACE cDNA Amplification kit (Promega, USA). The manufacturer’s protocol was followed for 5′ end analysis. In brief, total RNA was isolated from leaf tissues at 7 DAG and ligated to a 5′ end RNA adaptor before being reverse transcribed using an oligo(dT) primer. The PCR reactions were performed using two pairs of gene specific reverse primers (Additional file 1: Table S5). The PCR products were gel purified, then cloned into the T-easy vector (Promega, USA) and sequenced.

### Vector construction and barley transformation

The miR393 over-expression and MIM393 construct were generated according to Bian et al. [32] and Bai et al. [33]. The clones used for vector construction were verified by sequencing and then were electroporated into *Agrobacterium tumefaciens* strain AGL1, which was used to transform Golden Promise barley plants. Barley transformation was performed using the *A. tumefaciens*-mediated co-cultivation approach described by Bai et al. [33]. The transformed plants were grown in a growth chamber (24°C, 16 h light/8 h dark) or under natural condition in 6-in. pots in a growth room (Zhejiang University).

## Results

### Overview of sRNAs expressed in barley embryos during seed maturation and germination

To obtain an overview of the miRNA expression profile in embryo during seed maturation and germination, we constructed sRNA libraries using samples at three different developmental stages: one time point in the grain storage phase during seed development (10 DPA), two time points during seed germination including 1 DAG (imbibition to radicle emergence), and 5 DAG (postgermination growth) (Fig. 1a). After removal of low-quality and adaptor contaminants (reads <18 nt) from the raw sequencing data, a total of 17,216,333; 17,729,069; and 20,330,126 clean reads were obtained from the libraries of 10 DPA, 1 DAG, and 5 DAG, respectively (Additional file 1: Table S1). There were two major populations of sRNAs according to their lengths (Fig. 1b). The peak of length distribution located at 24 nt accounted for 69.44%, 19.09% and 32.5% in the three libraries respectively, indicating that siRNA-associated regulation is involved in heterochromatin modification and RNA-directed DNA methylation occurring at these stages. This is consistent with a previous study in rice which showed that filling or developing grains contained the most abundant 24-nt small RNAs [15]. The 21-nt small RNAs were the second most abundant population present in the three libraries, representing 8.48%, 16.48%, and 18.05% of the total reads, respectively. After getting rid of reads which could be annotated as rRNA, repeats, exons or introns, the remaining reads were used for miRNA prediction.

### Identification of known and novel miRNAs expressed in barley embryo

Through bioinformatics analysis, 1324 known miRNA families and 448 novel miRNA candidates were found to be expressed at total RPM (reads per million) up to 1 (Additional file 2: Table S2). Known miRNAs were identified through alignment to the sequences already registered in miRBase (Release 21), whereas novel miRNAs were predicted according to the characteristic hairpin structures of their precursors. As is shown in the Venn diagram, 531 conserved miRNA families and 40 novel miRNA candidates were detected in the embryo of developing and imbibed seeds at the three stages we tested. A total of 152 and 127, 98 and 89, and 179 and 118 known and novel miRNA families were found to be expressed specifically in embryos at 10 DPA, 1 DAG and 5 DAG, respectively.

**Fig. 2:**
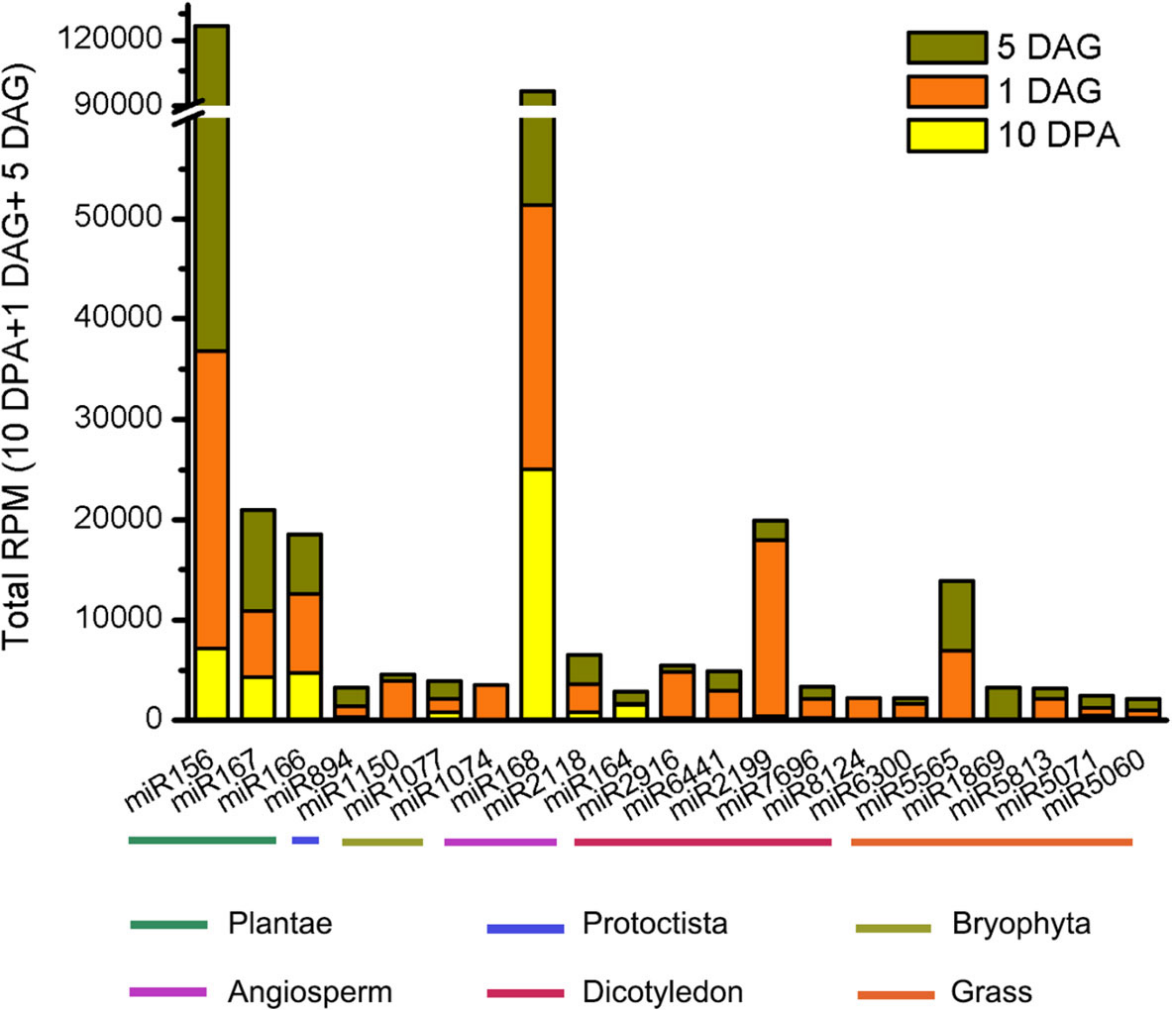
Summary of highly expressed microRNAs in barley embryos. miRNA families were grouped based on their conservation level across plant kingdom according to the miRNA information in miRBase (Release 21). The total number sequences from three libraries is presented in reads per million (RPM)

The top 21 highly expressed known miRNA families by total RPM (> 2000) were grouped according to their degree of conservation across the plant kingdom (Fig. 2). Among them, four miRNAs including miR156, miR167, miR166, and miR894 are conserved in all plant species registered in miRBase; three miRNAs (miR168, miR2118, and miR164) are conserved among angiosperm; six miRNAs (miR2916, miR6441, miR2199, miR7696, miR8124, and miR6300) are detected only in dicotyledons; and five miRNAs (miR5565, miR1869, miR5813, miR5071, and miR5060) seem to be conserved families across grass families. Notably, we found that miR5565 and miR2199 were significantly and highly expressed in barley embryos during seed germination, whereas miR156, miR166, miR167, and miR168 accumulated in both the seed development and germination stages (Fig. 2).

### Differential expression of miRNAs in embryo during seed development and germination

**Fig. 3:**
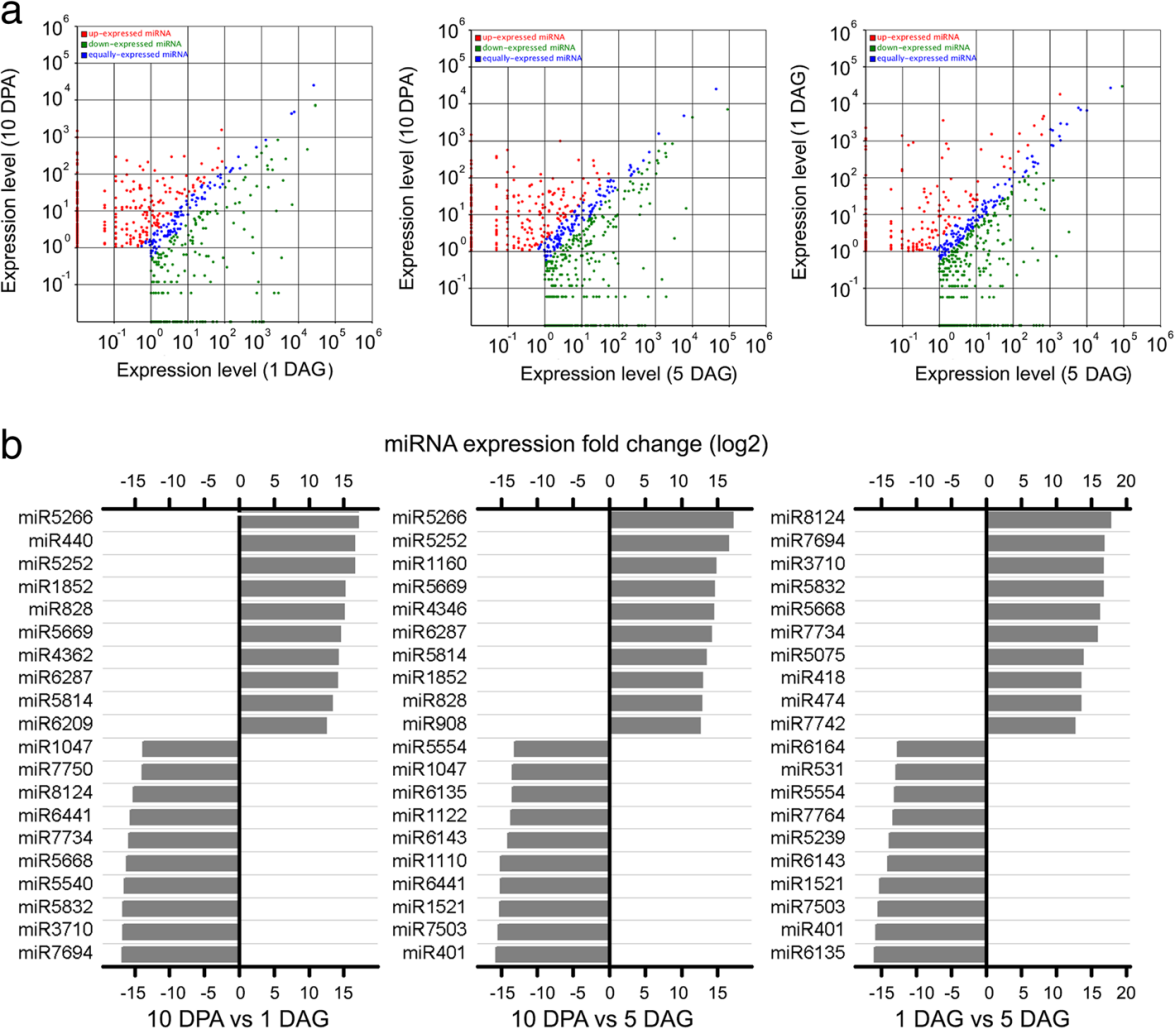
Differentially expressed miRNAs between seed maturing and germination stages. **a**: Scatter plot showing differentially expressed known miRNA between two different samples. Each point in the figure represents a miRNA. The X axis and Y axis show expression level in two samples respectively; **b**: Differential expressed miRNAs between two samples

The expression levels of known miRNAs were normalized to TPM (transcript per million). Several miRNAs were found to change dramatically in embryos for each pair-wise comparison among the three development stages (Fig. 3a). Among them, significantly differentially expressed miRNAs between two samples with a fold change greater than 12 (*p* value <0.01) are shown in Fig. 3b. In the filling stage, miR5266, miR5252, miR5669, miR1852 and miR5814 were much more highly expressed than in the germination stage. On the contrary, miR6441 and miR1047 had elevated their expression levels at the early stage of seedling growth. miR6135 and miR401 were only expressed highly at 5 DAG, whereas miR5832 and miR7734 were expressed preferentially at 1 DAG.

### Target prediction and function analysis of miRNAs

The target genes of miRNA candidates were predicted using the barley Unigene library (Esembl), Mireap, and psRNATarget (Additional file 3: Table S3). Furthermore, we searched for evidence from available degradome data from NCBI to select miRNAs that could result from cutting of the target mRNAs. A total of 40 genes targeted by 16 conserved miRNA families were identified (Table 1). Most of these targets are annotated as transcription factor-coding genes, including SQUAMOSA promoter-binding protein-like genes (*SPLs*) targeted by miR156, MYB transcription factors (*MYBs*) targeted by miR159, auxin response factors (*ARFs*) targeted by miR160, and TCP family transcription factors (*TCPs*) targeted by miR319. miR444 is predicted to target MLOC_16182, a gene encoding a RING finger protein; miR5051 targets MLOC_57965 encoding a serine/threonine protein kinase; and miR7757 targets MLOC_17471 encoding a putative NBS-LRR disease resistance protein. These miRNA-target pairs are reported here in barley for the first time. The cleavage signature for miR9863 in MLOC_24045 encodes a putative disease resistance protein (CC-NBS-LRR class) family member, consistent with a previous study [33]. As for novel miRNAs, only three novel miRNA-mediated cleavage sites in four genes have been confirmed by degradome sequencing data (Table 2).

**Table 1:** Target prediction and degradome analysis of known miRNAs

miRNA	Target CDS_ID	Target annotation	Binding site on CDS	PARE_cut sites
miR156	AK356077	Squamosa promoter-binding protein	839–858	849
	AK374598	Squamosa promoter-binding protein	860–879	870
	MLOC_11199.2	Squamosa promoter-binding-like protein 11	1154–1173	1164
miR159	AK251726.1	MYB transcription factor	962–982	973
	AK370348	Predicted protein	1009–1028	948 and 1019
miR160	MLOC_64795.1	Auxin response factor 17	1329–1349	1340
	MLOC_69988.1	Auxin response factor 10	717–737	728
miR165	MLOC_44268.1	MATE efflux family protein	159–179	170
	AK362009	Class III homeo domain-leucine zipper	578–598	589
	AK364215	Class III homeo domain-leucine zipper	639–658	649
	MLOC_58644.1	Class III homeo domain-leucine zipper	980–1000	991
miR166	AK362009	Class III homeo domain-leucine zipper	578–598	589
	AK364215	Class III homeo domain-leucine zipper	638–658	649
	AK364215	Class III homeo domain-leucine zipper	640–658	649
	AK365312	Class III homeo domain-leucine zipper	563–583	574
	MLOC_44268.1	MATE efflux family protein	159–179	170
	MLOC_58644.1	Class III homeo domain-leucine zipper	980–1000	991
miR167	MLOC_51932.2	Auxin response factor 30	2343–2363	2354
	MLOC_58330.2	Auxin response factor 18	2528–2549	2540
	MLOC_63938.1	Auxin response factor 9	2402–2423	2414
miR171	AK371946	GRAS family transcription factor	1090–1109	1100
miR172	AK355002	AP2-like ethylene-responsive transcription factor	1214–1234	1225
miR319	AK370348	TCP family transcription factor containing protein	1009–1028	1019
	MLOC_63989.1	TCP family transcription factor containing protein	976–995	986
miR360	MLOC_69988.1	Auxin response factor 19	717–737	728
miR393	AK355927	Auxin signaling F-box 2	1560–1580	1571
	AK374984	Auxin signaling F-box 3	1974–1994	1985
miR396	MLOC_64055.4	Growth-regulating factor 2	375–395	386
	AK250947.1	Growth-regulating factor 1	480–500	491
	AK353813	Growth-regulating factor 1	387–407	398
	AK376404	Growth-regulating factor 5	417–437	428
	MLOC_67201.4	Growth-regulating factor 1	741–761	752
	MLOC_80060.1	Growth-regulating factor 1-like	507–527	518
	MLOC_12347.1	Pre-mRNA-processing protein 45	1438–1458	1449
	AK375237	Nascent polypeptide-associated complex subunit alpha-like protein	184–204	195
miR444	MLOC_16182.1	RING finger protein	1035–1055	1046
miR5051	MLOC_57965.3	Serine/threonine protein kinase	595–615	606
miR7757	MLOC_17471.2	NBS-LRR disease resistance protein, putative	194–215	206
miR9863	MLOC_24045.1	Disease resistance protein (CC-NBS-LRR class) family	1259–1280	1271

**Table 2:** Novel miRNA-target pairs in barley based on the degradome data sets

miRNA	Sequencing	cDNA_ID	Target annotation	miRU start-ending	PARE_cutsites
novel-mir-119	TCTTGACCTTGCAAGACCTTT	AK362090	ARF 3	1362–1382	1373
		AK369226	ARF 3	423–443	434
novel-mir-400	CGACGAGTCGGACGCGTCGAGCA	MLOC_53497.2	Predicted protein	587–609	600/733
novel-mir-205	TCACAGATAATGGTGGCCCCTG	MLOC_54213.1	Predicted protein	389–410	401

All the potential target genes were functionally annotated by ontology analysis. The predicted targets were classified into three main categories: biological processes, cellular components, and molecular functions. Of these, cell, cell part, and organelle part in cellular components, molecular binding, catalytic activity, enzyme regulator activity, molecular transducer activity, metabolic processes, binding, catalytic activity, transcription regulation in cellular components and response to stimulus in biological processes were the most observed categories (Additional file 4: Figure S1), indicating that miRNAs might play roles in diverse and important biological processes.

### miRNAs involved in phytohormone signalling during seed development and germination

**Fig. 4:**
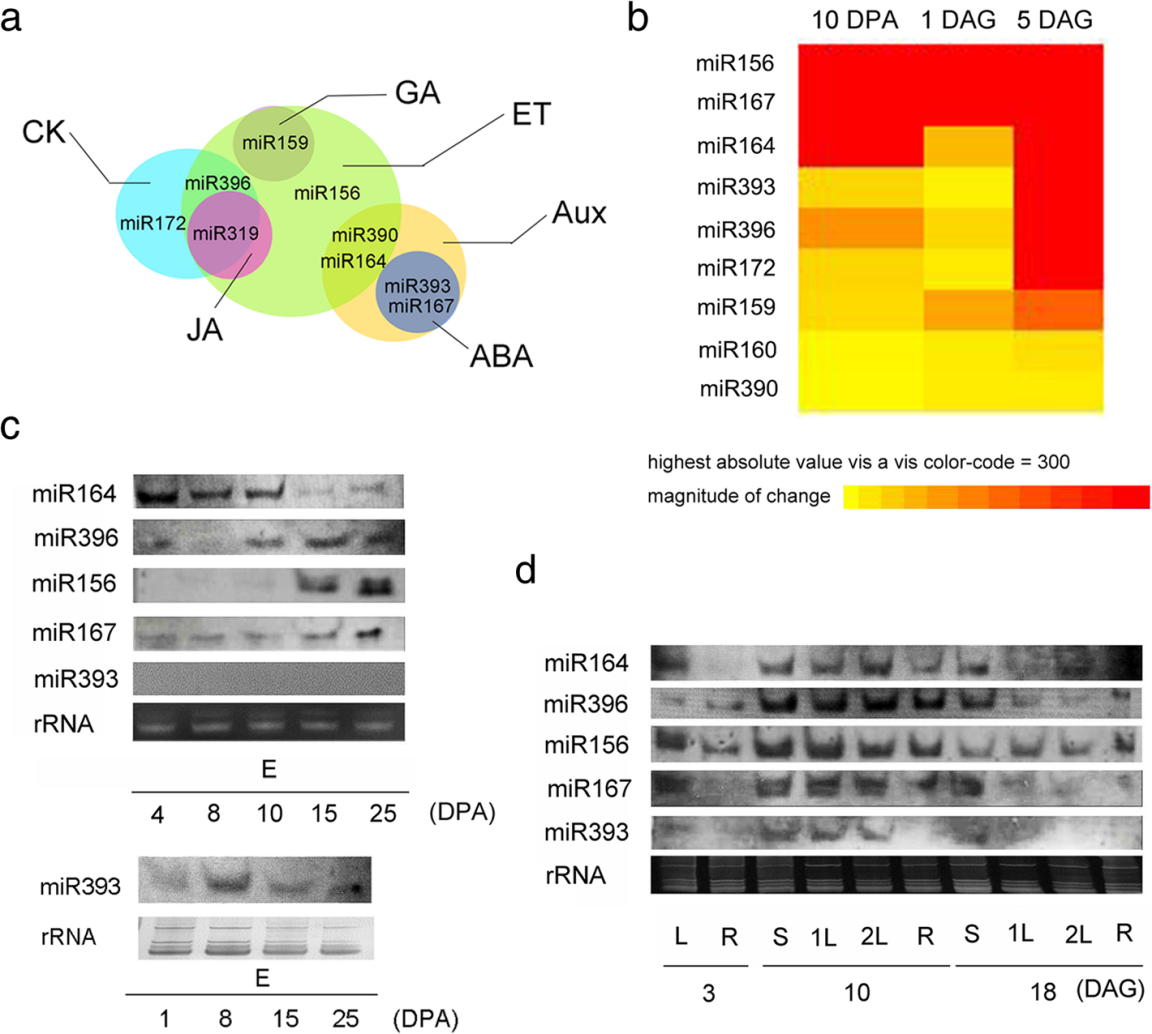
Phytohormone-related miRNAs involved in seed maturation and seed germination processes. a: Overview of the miRNA-mediated regulations in phytohormone crosstalk through KEGG pathway analysis; b: sRNA sequencing data showing the expression of miRNA during seed development and germination. The expression levels are visualized by BAR HeatMapper. Numbers beneath the heat map indicate the relative expression intensities, and the higher expression intensities are indicated by more reddish colors; c: Northern blot analysis of miRNA levels in embryos during seed development. 30 μg and 50 μg (for miR393) total RNA was loaded, and rRNA was stained by EtBr as loading control. E, embryo; d: Northern blot analysis of miRNA levels during early seedling growth. Total RNA was isolated from tissues in different development stages. L, leaf; R, root; S, stem; 1 L, first leaf; 2 L, second leaf

KEGG pathway analysis was performed for the target gene candidates. Nine miRNAs were predicted to be involved in phytohormone signalling pathways (Fig. 4a). Among them, miR156, miR159, miR390, miR164, miR396, and miR319 were predicted to regulate the ethylene pathway, miR159 regulates GA signalling; and miR172, miR396, and miR319 are likely to be associated with cytokinin signalling. Four miRNAs including miR393, miR390, miR164, and miR167, seem to be involved in the auxin pathway, whereas miR393 and miR167 regulate genes in the ABA pathway. miR319 is also predicted to be associated with the jasmonic acid pathway. Most of these miRNAs are involved in at least two phytohormone pathways, suggesting that the control of these two development stages involves cross-talk among five key phytohormones.

To determine the abundance of these miRNAs, the accumulation of nine known miRNA families was compared through sequencing data analysis combined with northern blot confirmation. As shown in Fig. 4b, miR156 and miR167 were expressed highly at the three developmental stages we tested; miR164 was expressed mainly at the seed development and postgermination stages; miR393, miR172, and miR396 were expressed preferentially in embryo at 5 DAG. The abundance of miR159 gradually increased during germination. The expression of five highly expressed miRNAs was further validated by northern blot analysis.. Overall, the results from northern blot analysis were in accordance with the sequencing data (Fig. 4c). In embryos during seed development, miR164 accumulated from 4 DPA to 10 DPA and decreased its level at the late period of seed development. miR396 and miR156 increased their abundance when seeds entered the maturation stage. The expression of miR167 seemed to be stable throughout the whole seed development period. miR393 levels varied dramatically, and could only be detected in embryos from 8 DPA after we increased the loading amount of RNA samples. During the early seedling growth stage, miR156, miR396, miR167, and miR164 increased their abundance in root, shoot, and leaf tissues, especially at 10 DAG. miR393 and miR167 were expressed highly in shoot and leaf tissues but not so in root tissues (Fig. 4d).

**Fig. 5:**
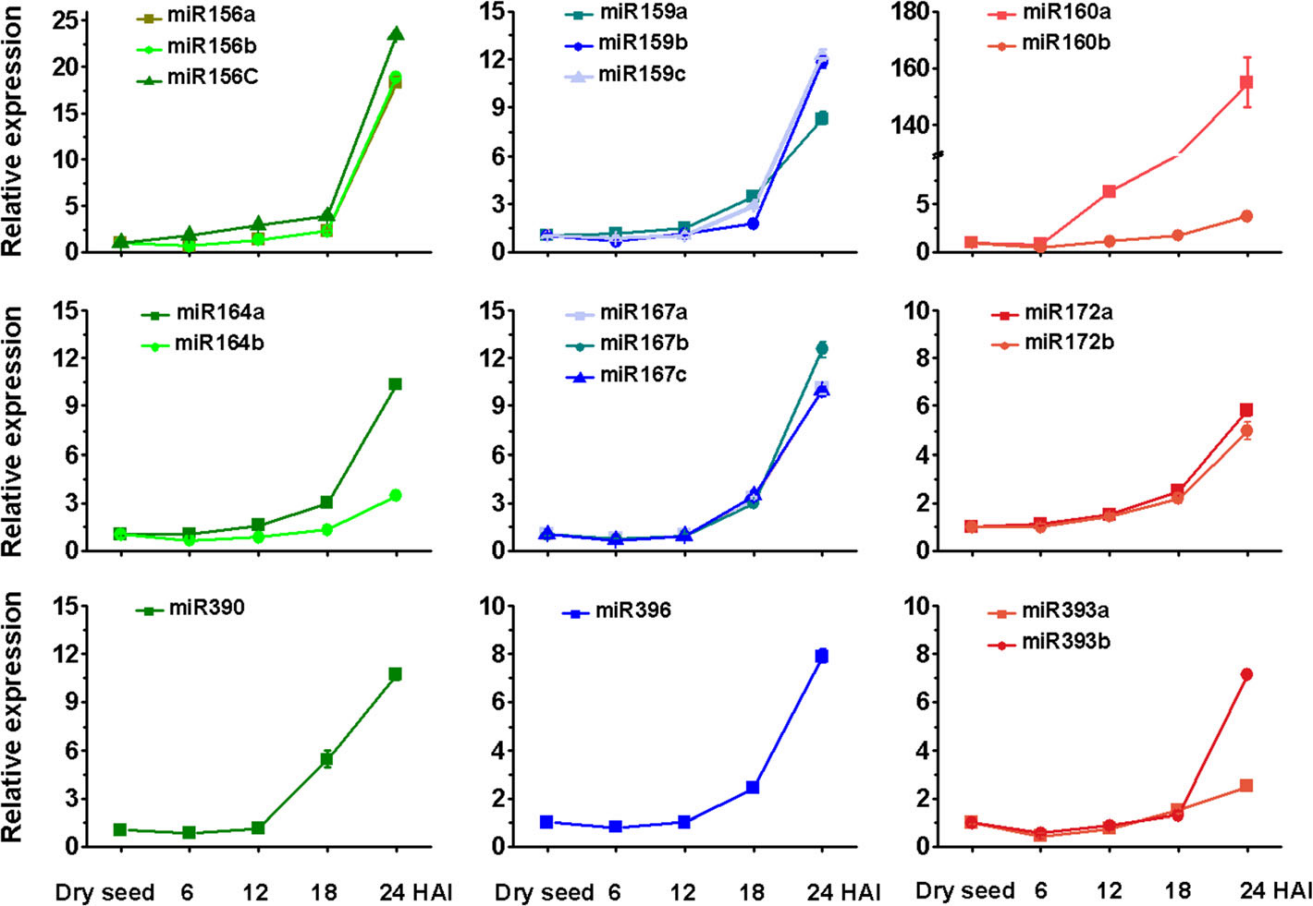
Real-time qRT-PCR detection of miRNAs during seed imbibition. Total RNA was isolated from embryo at different time points in germination stage including dry seed, 6 HAI (6 h after imbibition), 12 HAI, 18 HAI, 24 HAI. The expression level in dry seed was set as 1.0. Error bars represent the SD from three independent experiments

To distinguish different members within known miRNA families, we performed qRT-PCR to detect the mature miRNA levels in embryo during seed imbibition. The first 24 h after imbibition is pivotal for seed germination, during which embryo cells rapidly switch from a quiescent state to a metabolically active state. All the miRNA members we tested increased their levels early in the 24-h imbibition, compared with their corresponding control (dry seed) (Fig. 5), suggesting that the expression of these miRNAs is dynamic and might participate in gene expression regulation during germination.

### Verification of miRNA-induced cleavages in the target mRNAs

**Fig. 6:**
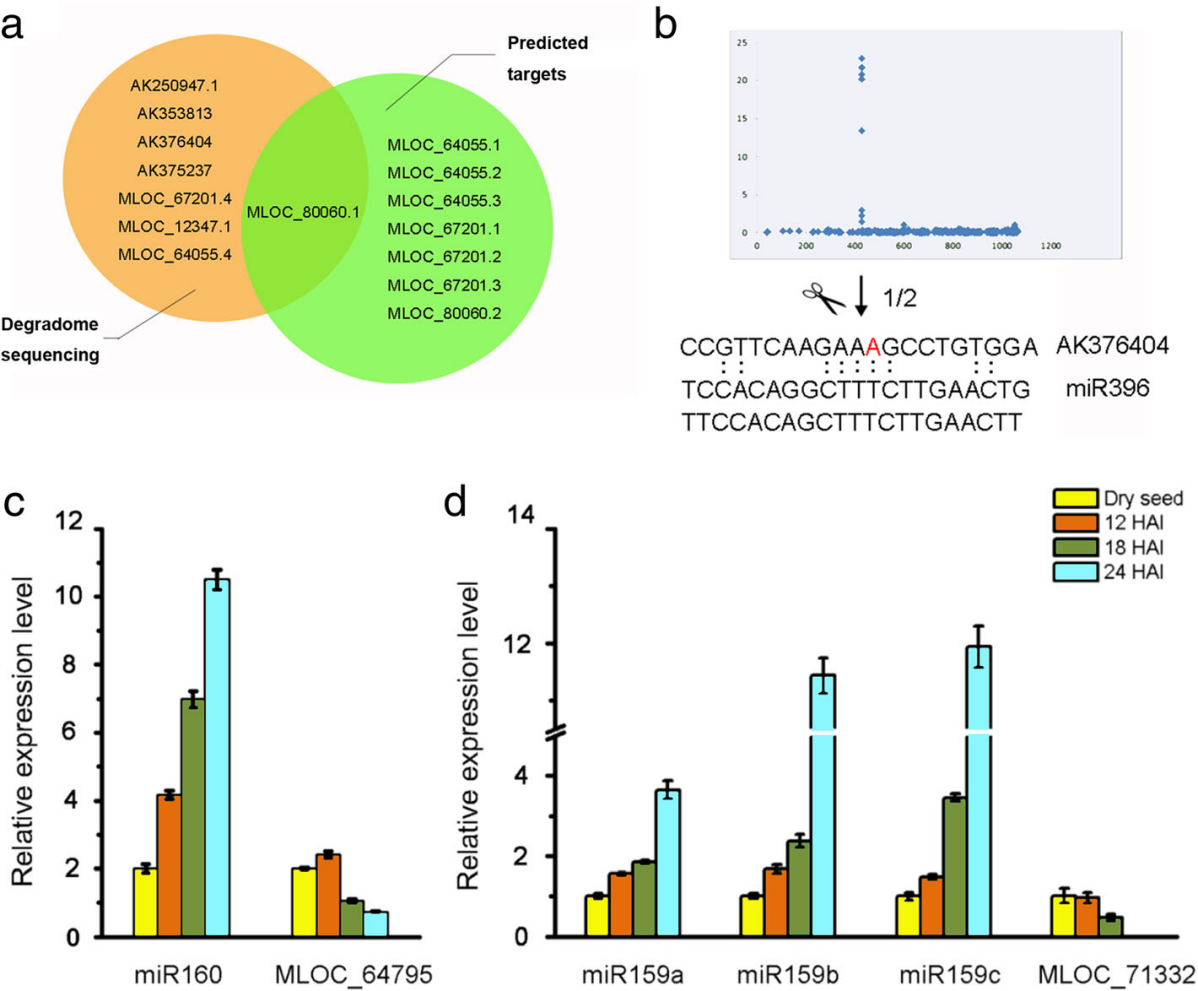
Validation of the target genes for Hvu-miR396, miR160 and miR159. a: Overlap between Hvu-miR396 targets predicted through psRNA Target software and degradome data analysis; b: Validation of the target genes for Hvu-miR396. Upper row shows the distribution of the degradome tag reads along the full length of the target mRNA sequence. Lower row shows modified 5′ RACE-PCR mapping of AK376404 mRNA cleavage sites. Arrows indicate miR396 cleavage sites. Numbers above depict the fraction of cloned products that terminated at the predicted cleavage site; c-d: Real-time qRT-PCR detection of miR160 (c), miR159 (d) and their target gene levels during seed imbibition. Total RNA was isolated from embryo at different time points in germination stage including dry seed, 12 HAI, 18 HAI, 24 HAI. The expression level in dry seed was set as 1.0. Error bars represent the SD from three independent experiments

The targets of phytohormone-associated miRNAs mentioned above are summarized in Additional file 1: Table S4 according to our study and previous studies. Moreover, we verified the target candidates for miR396 through bioinformatics prediction coupled with degradome sequencing data-based validation (Fig. 6a). Only one gene MLOC_80060 was found to overlap from the results of the two methods. To further examine the cleavage sites in target transcripts, we conducted 5′-RACE using RNA extracted from leaves of the 1-week-old wildtype plants (WT). We detected two major cleavage products from transcripts of AK376404 after nested PCR and gel electrophoresis. Sequencing of 5′-RACE clones revealed one cleavage site in transcripts of AK376404 (Fig. 6b), which is annotated as a *GRF* transcriptional factor.

Based on the information from target genes, the expression levels of miR160, miR159, and their targets were selected for detection using qRT-PCR (Fig. 6c, d). The transcript levels of MLOC_64795 (targeted by miR160) and MLOC_71332 (targeted by miR159) exhibited a gradually declining trend in the embryos of germinating seeds, whereas miR160 and miR159 increased their abundance when seeds started germinating.

### miR393-mediated auxin signalling regulation affects seed development and germination

**Fig. 7:**
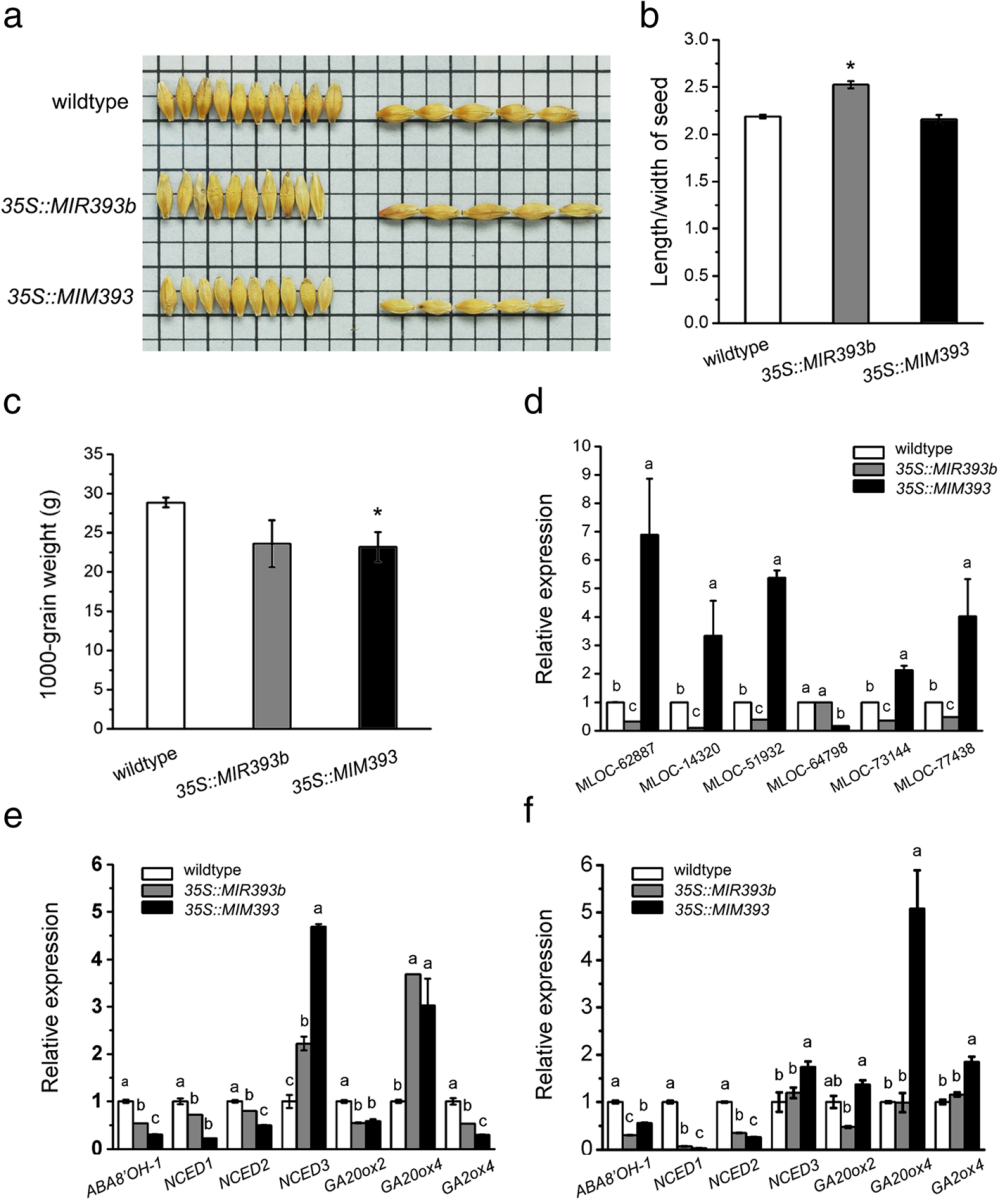
miR393-mediated auxin signalling regulation affects grain phenotypes and phytohormone cross-talk during seed germination. **a**: Seed phenotype of wildtype, *35S::MIR393b* and *35S::MIM393* transgenic barley; **b**: Length-width ratio of seeds in wildtype, *35S::MIR393b* and *35S::MIM393* transgenic barley; **c**: 1000-grain weight of wildtype, *35S::MIR393b* and *35S::MIM393* transgenic barley; Asterisks indicate significant difference compared with wildtype. *, *P* < 0.05 (Student’s *t*-test); **d**: Relative expression levels of auxin-responsive genes in wildtype, *35S::MIR393b* and *35S::MIM393* transgenic barley. Total RNA was isolated from 3 d-old seedlings. The expression level in wildtype was set as 1.0. Error bars represent the SD from three independent experiments; **e**-**f**: Relative expression level of ABA and GA-related genes in wildtype, *35S::MIR393b* and *35S::MIM393* transgenic barley. Total RNA was isolated from embryo and cotyledon (**e**) and root (**f**) in 3 d-old seedlings. The expression level in wildtype was set as 1.0. Error bars represent the SD from three independent experiments. Different letters represent significantly different values at *P* < 0.05 (Duncan’s multiple range test)

In our previous work, we identified two miR393 family members in barley and confirmed two target genes, *HvTIR1* (MLOC_9864) and *HvAFB* (MLOC_56088) through a modified form of 5′-RACE (rapid amplification of cDNA ends) as well as through degradome data analysis [34]. To investigate the biological function of miR393/target modules in seed development, we generated transgenic barley plant overexpressing miR393 (*35S::MIR393b*) or inhibiting miR393’s activity through an artificial miRNA target mimics strategy (*35S::MIM393*). Through northern blot and qRT-PCR analysis, we showed that miR393 negatively regulates the transcript levels of its two target genes (*HvTIR1* and *HvAFB*) [34]. Moreover, we found that miR393 affects barley seed size and shape. As shown in Fig. 7a and b, compared with wildtype, the length/width ratio of the seed was greatly increased in *35S::MIR393b* plants, although the 1000-grain weight did not change significantly. In *35S::MIM393* plants, only the 1000-grain weight was significantly decreased (Fig. 7c).

Because miR393 targets two genes encoding the auxin receptor TIR1/AFBs, we then detected the expression of auxin signalling-related genes in these transgenic lines. This analysis showed that the transcripts of two *ARF* members (MLOC_77438 and MLOC_73144), one *SAUR* family member (MLOC_62887) and one *AUX/IAA* family member gene (MLOC_14320) were upregulated by more than two times in 3-d-old seedlings of the MIM393 line, and downregulated in miR393 over-expression lines (Fig. 7d), indicating that the phenotypic changes in seed development might be due to the effect of miR393 on auxin response.

We then examined variation in gene expression in these transgenic lines, including for an ABA catabolic gene (*ABA8’OH-1*), ABA biosynthesis genes (*NCED1, NCED2, NCED3*) and three GA biosynthesis-associated genes (*GA20ox2, GA20ox4, GA2ox4*), all in embryo, cotyledon, and root samples during the germination stage. The analysis showed that these genes displayed differential expression in miR393 misexpression transgenic lines (Fig. 7e, f), indicating that miR393 influences ABA and GA signal pathways as well.

## Discussion

So far, sRNA and degradome sequencing have been performed to identify miRNAs in seeds of crop plants including rice, maize, wheat and barley [18–20, 35]. However, detailed spatial and temporal studies of miRNA-mediated regulation are still limited. In this study, we investigated miRNA-mediated gene regulation in barley embryo during seed maturation and germination. The three samples we selected included 10 DPA, 1 DAG, and 5 DAG. The sample from 10 DPA represents transition of the seed to a storage phase characterized by dramatic transcriptional changes to mobilize energy resources and to differentiate the tissues that will constitute the mature grain. The samples from 1 DAG and 5 DAG represent the germination and post-germination stages, respectively. Via high-throughput sequencing and qRT-PCR detection, we revealed dynamic features of the regulatory network mediated by miRNAs in embryos during barley grain development and germination.

### Conserved and barley-specific miRNAs highly expressed in embryo tissues

According to bioinformatics analysis, we found that miR156 was the most highly expressed miRNA in embryos of developing and germinating seeds. miR156 is reported to be one of the most abundant miRNAs conserved among nearly all land plants including mosses [36]. In *Arabidopsis*, miR156 regulates the juvenile-to-adult phase and floral transition through targeting *SPLs* [37–39]. In rice and maize, miR156, together with miR172, acts as a regulator of inflorescence and tiller development [40–42]. Regulation of *OsSPL14* by OsmiR156 defines the ideal plant architecture in rice, whereas *OsSPL16*, another target of miR156, is involved in a regulatory module determining grain shape and can be modulated to improve rice yield and grain quality [43, 44]. In this study, we showed that miR156 exhibited a dynamic expression pattern during seed development and germination. miR156 preferentially accumulated in embryos at 15 DPA and 25 DPA (Fig. 4c) and displayed an increasing trend in the early seedling growth stage (Figs. 4d and 5). Through analysis of degradome sequencing data, three *SPL* gene family members including AK356077, AK374598 and MLOC_11199 were predicted to be the target genes of miR156 (Table 1). miR166, which is predicted to target five transcription factors belonging to the Class III homeodomain-leucine zipper (HD-ZIP III) family (Table 1 and Additional file 1: Table S4), was expressed at a very high level in the three samples we tested. miR166/HD-ZIP III module was reported to regulate cell differentiation in root and shoot apical meristems of *Arabidopsis* [45, 46]. This indicates that miR156 and miR166-mediated repression of transcriptional factors might be conserved mechanisms to modulate cell differentiation during seed development and germination.

Another conserved miRNA family, miR894, was found to accumulate at a very low level when using samples of an entire barley seed (0–15 DPA) [20]; however, we showed that miR894 was expressed highly both in embryo of developing and germinating seeds. Moreover, the expression level was elevated more than three times at 5 DAG, when compared with that at 1 DAG (Fig. 2, Additional file 2: Table S2). miR5071, which has been identified only in barley, accumulated at 730; 1234; and 2484 RPM at 10 DPA, 1 DAG, and 5 DAG, respectively (Additional file 2: Table S2). The target of miR5071 was predicted to be an *OsMLA10-like* gene [20]. The expression of miR5071 was mostly detected in embryo, whereas *OsMLA10-like* transcript was expressed highly in endosperm dissected from the caryopsis at 15 DPA [20]. In barley, R gene *MLA10* was reported to act as a receptor of fungal infection by recognizing avirulence proteins and confer resistance against powdery mildew fungus [47]. This suggests that miRNAs participate in defence response regulation during these two developmental stages.

Other noteworthy miRNAs, which are highly expressed in embryos, include miR5565 and miR2199. miR5565 has been identified in *Sorghum bicolor* [48] and is predicted to target a RING/U-box superfamily protein in barley [20]. As for miR2199, which has previously been reported only in common bean *Phaseolus vulgaris* and switchgrass [49], its expression and target genes in barley still need further confirmation. Overall, our results demonstrate that both conserved and barley-specific miRNAs contribute to the gene regulation in the embryo during seed development and germination.

### miRNA-mediated hormone signalling regulation in embryos

ABA and GA are two major players determining seed maturation, dormancy and germination. ABA can be synthesized during seed maturation in the maternal tissue and the embryo, and its content decreases rapidly during imbibition [50, 51]. GA is synthesized and stored at least in the embryo and is released during imbibition to trigger the synthesis and secretion of hydrolytic enzymes in the aleurone for endosperm storage product mobilization [52]. In the present work, we showed that miRNAs are regulators of ABA and GA signalling during grain development and germination. GA-related miRNA miR159 was mainly expressed during the germination stage (Figs. 4 and 5), which targets a gene (AK251726/MLOC_71332) encoding a *MYB* transcription factor and another predicted protein (Table 1). qRT-PCR analysis showed that there was an inverse correlation between miR159a/b/c and their target MLOC_71332 (Fig. 6), implying that miR159-mediated target repression occurs during the first 24 h after imbibition.

miR168, which has been found to respond to salt, drought, and cold stresses or ABA treatment in previous studies, was the second most highly expressed miRNA in embryo tissue. In *Arabidopsis*, it is established that transcriptional regulation of *MIR168a* and *ARGONAUTE1* homeostasis plays a critical role in abscisic acid and abiotic stress responses [53]. Although we could not identify miR168-mediated target sliced products because of the different samples used in sRNA sequencing and degradome analysis, miR168 was predicted to target *AGO1* based on degradome sequencing using boron-treated barley seedling samples [54]. In addition, we found that miR168 accumulated to a high level in the three samples tested, suggesting that miR168 might be influenced by ABA in seed and probably participate in stress response during seed desiccation and germination.

Auxin, which acts as a versatile trigger in many developmental processes, also plays a critical role in seed development and germination [8]. A total of five miRNAs were found to be related to auxin signalling in our study, which included miR160 (targeting *ARF10* and *ARF17*), miR167 (targeting *ARF30, ARF18,* and *ARF9*), miR360 (targeting *ARF19*) and miR393 (targeting *AFB2* and *AFB3*) (Table 1). Two independent reports using an auxin signaling reporter, *DR5:β-glucuronidase (GUS)* showed that auxin-dependent responses increase during embryogenesis and remain present in germinating seeds [25, 55]. Accordingly, we observed differential expression of these miRNAs through northern blot and qRT-PCR analysis. During seed development, miR393 accumulated to a high level from 8 DPA, and miR167 exhibited a expression pattern opposite to that of miR164, although both the latter miRNAs seem to regulate *ARF* genes involved in auxin signalling (Fig. 4c). During the germination process, miR160, miR167, and miR393 were all upregulated within the first 24 h after imbibition (Fig. 5). We proposed that these miRNAs might be strongly induced by endogenous auxin, and affect auxin signalling in different ways.

The importance of these miRNAs in seed development and germination is supported by previous studies in other plant species. Repression of *ARF10* by miR160 is thought to be critical for seed germination and post-germination stages [25]. miR160-directed regulation of *Arabidopsis ARF17* is essential for proper development and modulates expression of early auxin response genes [25]. Plants expressing a miRNA-resistant version of *ARF17* altered accumulation of auxin-inducible *GH3-like* mRNAs and had dramatic developmental defects, including the embryo, floral organ development, and sterility [56]. Rice transgenic plants expressing an OsmiR160-resistant version of *OsARF18* (*mOsARF18*) exhibited pleiotropic defects in growth and development, such as dwarf stature, rolled leaves and small seeds with reduced starch accumulation [57]. *Arabidopsis* miR167 controls patterns of *ARF6* and *ARF8* expression and regulates both female and male reproduction [32]. miR393, which targets F-box genes encoding TIR1/AFBs auxin receptors, is implicated to regulate seed development in rice. Transgenic rice plants over-expressing miR393 displayed reduced seed size, and the out glume of the spikelet had an abnormal shape and failed to close [58].

In this study, we showed that over-expression of miR393 greatly enhanced the length-width ratio of the seed, whereas over-expression of MIM393 significantly decreased the 1000-grain weight. Combined with the observation that the differential expression of auxin response genes such as *GH3*, *AUX*/*IAA* and *ARFs* in transgenic lines (Fig. 7d), this demonstrates that miR393 affects seed development through negative regulation of the auxin response, although the detailed mechanism underlying phenotypic changes in miR393 misexpression lines still need further study to elucidate.

### Interplay between auxin and ABA/GA

The cross-talk of diverse hormonal signals in seed development, seed dormancy, and germination has been reported in many previous studies. For example, auxin promotes dormancy and inhibits germination through stimulation of ABA signalling by inducing *ARF*-mediated *ABI3* activation in *Arabidopsis* [9]. ABA represses embryonic axis elongation during seed germination, also potentiating auxin signalling [59]. The interactions of different hormone signals also involve miRNAs. In germinating *Arabidopsis* seeds, miR159 accumulation can be induced by ABA in an *ABI3*-dependent fashion. The targets of miR159 such as *MYB101* and *MYB33* encode transcription factors functioning as positive regulators of ABA responses, suggesting that ABA-induced accumulation of miR159 is a homeostatic mechanism to desensitize hormone signalling during seedling stress responses [41].

In this study, we found that nine miRNAs expressed in embryo regulate seed development and germination through a complex interaction of phytohormone signalling pathways based on the KEGG analysis of target genes (Fig. 4a). Moreover, we showed that miR393 regulated auxin signalling and hormone interaction. Two genes targeted by miR393 encode TIR1/AFBs auxin receptors in barley, which are components of the Skp1-Cullin1-F-box protein ubiquitin ligase complexes. The complex regulates auxin signalling via the release of ARFs from Aux/IAA (auxin/indole-3-acetic acids)-mediated heterodimerization [60, 61]. miR393 is now regarded as an important regulator of auxin signaling [62] and it affects various development processes including seed development (Fig. 7), root development and root growth response to toxic Al in barley [34]. qRT-PCR detection of auxin-related genes during germination and early seedling growth indicated that miR393 affected the expression of three putative *ARF* genes and two early auxin responsive genes in miR393 misexpression lines (Fig. 7d). This was consistent with previous data in *Arabidopsis* and barley [34, 62] and supported the notion that miR393 has a major influence on auxin homeostasis. Our data also showed that four ABA-related gene (*ABA8’OH-1* and *NCED1, 2,* and *3*) and three GA-associated genes (*GA20ox2, GA20ox4,* and *GA2ox4*) were differentially expressed in miR393 misexpression lines (Fig. 7e, f), compared with their wildtype controls. We supposed that the effect of miR393-mediated regulation on ABA and GA homeostasis might be complex, dependent on the different isoforms of related gene families and specific tissue fractions where they are expressed. Another possibility is that the variation in auxin response might influence the GA/ABA balance, rather than GA or ABA signalling alone, in the germination process. Further investigation to identify the critical factors that connect different hormone pathways will help us to better understand the molecular mechanism of hormone cross-talk.

## Conclusions

Our study performed a detailed analysis of miRNA expression profiles and miRNA-target pairs in barley embryo and provided evidence for miR393-mediated regulation of auxin response and its interaction with the ABA and GA pathways during seed development and germination.

## Additional files

Additional file 1: Table S1. Summary of data cleaning and length distribution of tags. **Table S4:** Highly expressed miRNAs and their target genes associated with phytohormone signaling pathways. **Table S5.** Primers and probes used in this study. (PDF 195 kb)

Additional file 2: Table S2. Known and novel miRNAs identified in barley embryo. (XLSX 196 kb)

Additional file 3: Table S3. Predicted targets of known miRNAs. (XLSX 678 kb)

Additional file 4: Figure S1. Functional distributions of predicted miRNA target genes expressed in the embryo. (PDF 151 kb)

## Abbreviations

ABA: Abscisic acid
AFB: Auxin-signaling F-box protein
ARF: Auxin response factors
Aux/IAA: Auxin/indole-3-acetic acids
ET: Ethylene
GA: Gibberellic acid
KEGG: Kyoto Encyclopedia of Genes and Genomes analysis
microRNA: miRNA
qRT-PCR: quantitative reverse transcription PCR
RACE: Rapid-amplification of cDNA ends
SCF: Skp1-Cullin-F-box
sRNA: small RNA
TIR1: Transport inhibitor response protein
