# Supplementary material for: microRNAs participate in gene expression regulation and phytohormone cross-talk in barley embryo during seed development and germination
Source: BMC Plant Biol. 2017 Sep 6;17:150. doi: 10.1186/s12870-017-1095-2 (PMC5586051; doi:10.1186/s12870-017-1095-2)
Supplement: Supplementary file 1 — Summary of data cleaning and length distribution of tags. Table S4: Highly expressed miRNAs and their target genes associated with phytohormone signaling pathways. Table S5. Primers and probes used in this study. (PDF 195 kb) [file 12870_2017_1095_MOESM1_ESM.pdf]

Table S1 Summary of data cleaning and length distribution of tags

Type	10 DPA		1 DAG		5 DAG	
	Count	Percent (%)	Count	Percent (%)	Count	Percent (%)
total_reads	17351184		17978337		20576026	
high_quality	17259618	100%	17878430	100%	20477866	100%
'adapter_null	9105	0.05%	4970	0.03%	7761	0.04%
insert_null	2073	0.01%	3740	0.02%	7874	0.04%
5'adapter_contaminants	24644	0.14%	29884	0.17%	91874	0.45%
smaller_than_18 t	6484	0.04%	110624	0.62%	39233	0.19%
polyA	979	0.01%	143	0.00%	998	0.00%
clean_reads	17216333	99.75%	17729069	99.16%	20330126	99.28%

Table S4 Highly expressed miRNAs and their target genes associated with phytohormone signaling pathways

miRNA	RPM			Predicted function for targets	Target ID	Verified target by RACE
	10 DPA	1 DAG	5 DAG			
**miR156**	7195.8	29590.2	90814	Squamosa promoter-binding-like protein	MLOC_11199	CL11193.Contig1_all [1]
				SBP family protein	MLOC_52321	U21_18637 [2]
				Uncharacterized protein	MLOC_61297	
				Uncharacterized protein	MLOC_62426	
**miR167**	4318.2	6581.1	10059.9	Auxin response factor 8	MLOC_51932.2	[3]
				Auxin response factor 8	MLOC_63938	[3]
				Auxin response factor 18	MLOC_58330.2	
				NEK5-like kinase	MLOC_5326	[3]
**miR164**	1570.7	83.5	1240.8	Uncharacterized protein	MLOC_53744	U21_9757 [2]
				NAC family protein	MLOC_64240	CL1686.Contig1_all [4]
				Uncharacterized protein	MLOC_65286	
				MYB family protein	MLOC_65555	
				Uncharacterized protein	MLOC_68221	
				Oxygen-evolving enhancer protein1, chloroplastic	MLOC_78630.1	[5]
**miR393**	48.9	14.3	587.4	Uncharacterized protein	MLOC_56088	MLOC_56088 [6]
				Uncharacterized protein	MLOC_9864	U21_7409 [6]
**miR396**	128.0	47.1	428.3	GRF family protein	MLOC_64055	AK376404
				GRF family protein	MLOC_67201	
				Uncharacterized protein	MLOC_80060	
**miR172**	48.8	24.6	413.0	AP2 family protein	MLOC_43041	HvAP2 （Cly1）[1, 7]
				AP2 family protein	MLOC_43575	
				AP2 family protein	MLOC_43830	Zeo1.b [8]
				AP2 family protein	MLOC_77763	
**miR159**	42.6	105.8	186.9	GAMYB	MLOC_6041	U21_3667 [2]
				Uncharacterized protein	MLOC_71332	CL32877.Contig1_all [1]

miRNA	RPM			Predicted function for targets	Target ID	Verified target by RACE
	10 DPA	1 DAG	5 DAG			
**miR159**				Uncharacterized protein	MLOC_74051	
**miR160**	8.9	23.2	36.3	Uncharacterized protein	MLOC_56664	
				Uncharacterized protein	MLOC_61554	
				Auxin response factor17	MLOC_64795	[3]
				Auxin response factor13	MLOC_67174	[3]
				Uncharacterized protein	MLOC_69988	
				Uncharacterized protein	MLOC_77438	
				Probable mitochondrial-processing peptidase subunit beta	MLOC_81236.1	[5]
				Probable plastid-lipid-associated protein 11,chloroplastic	MLOC_80837.2	[5]
**miR390**	6.4	21.4	21.7	Uncharacterized protein	MLOC_4946	
				Predicted protein	MLOC_61478	
				Predicted protein	MLOC_65593	
				Uncharacterized protein	MLOC_80454	

References

1. Ozhuner E, Eldem V, Ipek A, Okay S, Sakcali S, Zhang B, Boke H, Unver T: **Boron stress responsive microRNAs and their targets in barley**. *PloS one* 2013, **8**(3):e59543.

2. Curaba J, Spriggs A, Taylor J, Li Z, Helliwell C: **miRNA regulation in the early development of barley seed**. *BMC plant biology* 2012, **12**:120.

3. Kruszka K, Pacak A, Swida-Barteczka A, Nuc P, Alaba S, Wroblewska Z, Karlowski W, Jarmolowski A, Szweykowska-Kulinska Z: **Transcriptionally and post-transcriptionally regulated microRNAs in heat stress response in barley**. *Journal of experimental botany* 2014, **65**(20):6123-6135.

4. Ozhuner E, Eldem V, Ipek A, Okay S, Sakcali S, Zhang B, Boke H, Unver T: **Boron stress responsive microRNAs and their targets in barley**. *PloS one* 2013, **8**(3):e59543.

5. Deng P, Wang L, Cui L, Feng K, Liu F, Du X, Tong W, Nie X, Ji W, Weining S: **Global Identification of MicroRNAs and Their Targets in Barley under Salinity Stress**. *PloS one* 2015, **10**(9):e0137990.

6. Bai B, Bian H, Zeng Z, Hou N, Shi B, Wang J, Zhu M, Han N: **miR393-Mediated Auxin Signaling Regulation is Involved in Root Elongation Inhibition in Response to Toxic Aluminum Stress in Barley**. *Plant & cell physiology* 2017.

7. Nair SK, Wang N, Turuspekov Y, Pourkheirandish M, Sinsuwongwat S, Chen G, Sameri M, Tagiri A, Honda I, Watanabe Y *et al*: **Cleistogamous flowering in barley arises from the suppression of microRNA-guided HvAP2 mRNA cleavage**. *Proceedings of the National Academy of Sciences of the United States of America* 2010, **107**(1):490-495.

8. Houston K, McKim SM, Comadran J, Bonar N, Druka I, Uzrek N, Cirillo E, Guzy-Wrobelska J, Collins NC, Halpin C *et al*: **Variation in the interaction between alleles of HvAPETALA2 and microRNA172 determines the density of grains on the barley inflorescence**. *Proceedings of the National Academy of Sciences of the United States of America* 2013, **110**(41):16675-16680.

Table S5 List of primers and probes used in this study

**Primers for qRT-PCR (5'-3')**	Forward	Reverse
Hvu-miR156a	TGACAGAAGAGAGTGAGCAC	
Hvu-miR156b	TGACAGAAGAGAGAGAGCAC	
Hvu-miR156c	TGACAGAAGAGAGCGAGCAC	
Hvu-miR159a	TTTGGATTGAAGGGAGCTCTG	
Hvu-miR159b	ACTGGATGACGCGGGAGCTAA	
Hvu-miR159c	TTGGACTGAAGGGTGCTCCCT	
Hvu-miR160a	GCGTGCAAGGAGCCAAGCATG	
Hvu-miR160b	TGCCTGGCTCCCTGTATGCCA	
Hvu-miR164a	TGGAGAAGCAGGGCACGTGCA	
Hvu-miR164b	TGGAGAAGCAGGGCACGTGCT	
Hvu-miR167a	TGAAGCTGCCAGCATGATCTA	
Hvu-miR167b	TGAAGCTGCCAGCATGATCTGA	
Hvu-miR167c	TGAAGCTGCCAGCATGATCTGC	
Hvu-miR172a	AGAATCTTGATGATGCTGCAT	
Hvu-miR172b	GGAATCTTGATGATGCTGCAT	
Hvu-miR390	AAGCTCAGGAGGGATAGCGCC	
Hvu-miR396	TCCACAGGCTTTCTTGAACTG	
Hvu-miR393a	TTCCAAAGGGATCGCATTGAT	
Hvu-miR393b	TCCAAAGGGATCGCATTGATC	
U6	TTGGACCATTTCTCGATTTGTGC	CCTTAGGGGACATCCGATAAAATTG
ACTIN	GCTGAGCGGGAAATTGTAAG	GATCATGGATGGCTGGAAGA
UBQ	GTAACCAGGCTCAGGAAG	TCTGGTTGTAGACATAGGTGA
MLOC_64795	GGCTGGGCACTGCAAGGTA	CACGGGTAAACAAACGCTAATAAAA
MLOC_71332	CTATCTCCACGAAGCCAACTCTA	GTTCCTCGGCGAAGCACA
HvSAUR (MLOC_62887)	TCTCCCTCCCTCCATTGTCGC	GTTCCTCGGCCATCTCACTTCG
HvAUX/IAA (MLOC_14320)	GGTCAAAACCCAACTGTGGC	CAAGAGGGTGGGGATAGGGA
HvARF (MLOC_51932)	TTTGGTGGCTCAGGTTTGC	TTCTGCTTGTTGGGTCATTCTC
HvARF (MLOC_64795)	GGCTGGGCACTGCAAGGTA	CACGGGTAAACAAACGCTAATAAAA
HvARF (MLOC_73144)	ACAACCCACAAACATATCGTCT	ATTCCTCGGTCTCGCACA
HvARF (MLOC_77438)	CACCCATCGCATCAAGAATCAGCA	CAGTATTGTCCGTCCGAAGAGCACC
HvABA8'OH1 (MLOC_4786)	AGCACGGACCGTCAAAGTC	TGAGAATGCCTACGTAGTG
HvNCED1 (MLOC_43893)	CCAGCACTAATCGATTCC	GAGAGTGGTGATGAGTAA
HvNCED2 (MLOC_18300)	CATGGAAAGAGGAAGTTGC	GAAGCAAGTGTGAGCTAAC
HvNCED3 (MLOC_61512)	TGACCTATTTCTCCTATG	CATCAGATTCAGATACAC
HvGA20ox2 (MLOC_56462)	AGCCAATAAGAGCACGAGAA	CCTCCTACATCTACTACATCATCT
HvGA20ox4 (MLOC_34543)	TAGCAAGGTGAAGTCGTA	TGGTATGATGTCTACAGGAA
HvGA2ox4 (MLOC_13981)	CAACAGGTGTGGTTACTAATG	GGACAAGGACAAGGACAA
**Primers for RACE**		
Adapter	GCUGAUGGCGAUGAAUGAACACUGCGUUUGCUGGCUUUGAUGAAA	
Outer Primer F	GCTGATGGCGATGAATGAACACTG	

Inner Primer F	CGCGGATCCGAACACTGCGTTTGCTGGCTTTGATG
AK376404 outer R	GGGACAGCGAGGCGATGGTTA
AK376404 inner R	CTCCTGCGACGGCTCAAAGTG
**Probe for Northern Blot** (lower-case letters for LNA)	
Hvu-miR393	AtCAaTGcGAtCCcTTtGGA
Hvu-miR396	AgTTcAAgAAaGCtGTgGAA
Hvu-miR156	GtGCtCAcTCtCTtCTgTCA
Hvu-miR167h	TcAGaTCaTGcTGgCAgCTtCA
Hvu-miR164	TgCAcGTgCCcTGcTTcTCcA
