# Supplementary material for: microRNAs participate in gene expression regulation and phytohormone cross-talk in barley embryo during seed development and germination
Source: BMC Plant Biol. 2017 Sep 6;17:150. doi: 10.1186/s12870-017-1095-2 (PMC5586051; doi:10.1186/s12870-017-1095-2)
Supplement: Supplementary file 4 — Functional distributions of predicted miRNA target genes expressed in the embryo. (PDF 151 kb) [file 12870_2017_1095_MOESM4_ESM.pdf]

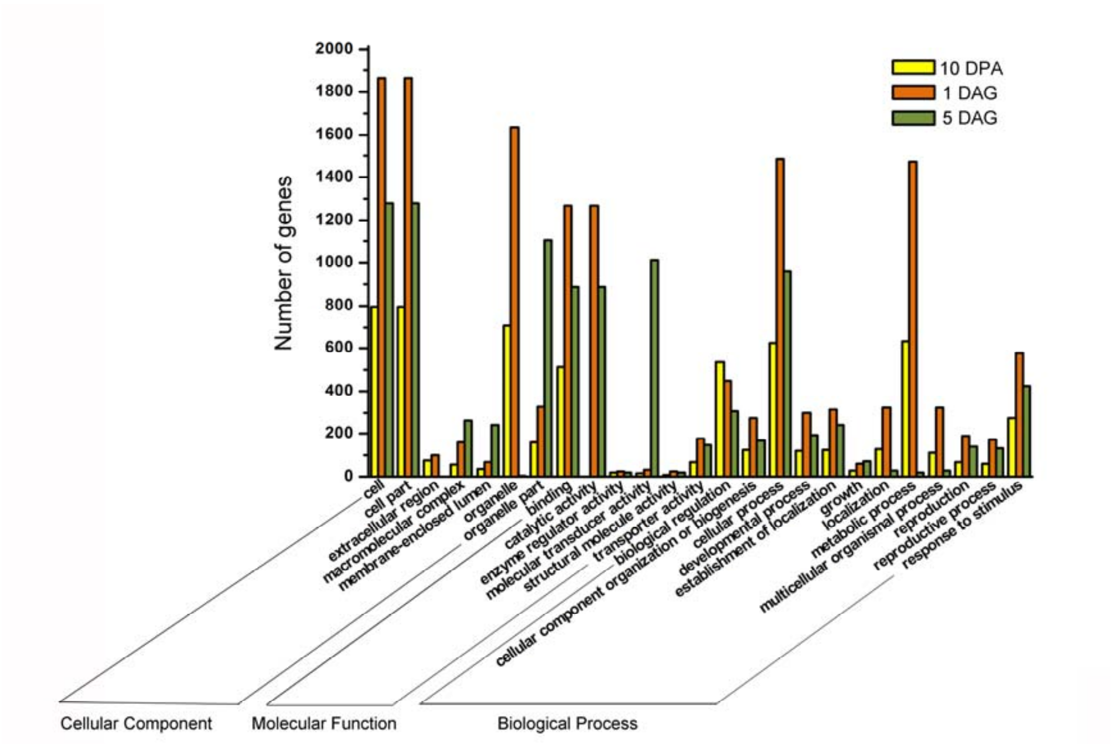

Figure S1 Functional distributions of predicted miRNA target genes expressed in the embryo. Potential target genes were functionally annotated by Ontology analysis, and were classified into three main categories: biological processes, cellular components, and molecular functions.
